# F69. MUSCARINIC M1 RECEPTOR SEQUENCE VARIATION AND GENERAL COGNITION

**DOI:** 10.1093/schbul/sby017.600

**Published:** 2018-04-01

**Authors:** Sean Carruthers, Kiymet Bozaoglu, Caroline Gurvich, Philip Sumner, Eric Tan, Elizabeth Thomas, Susan Rossell

**Affiliations:** 1 Swinburne University; 2 Bruce Lefroy Centre for Genetic Health Research, Murdoch Children’s Research Institute, University of Melbourne; 3 Monash Alfred Psychiatry Research Centre, The Alfred Hospital and Monash University; 4 Swinburne University, Monash Alfred Psychiatry Research Centre, The Alfred Hospital; 5 Monash Alfred Psychiatry Research Centre, Brain and Psychological Sciences Research Centre, Swinburne University; 6 Monash Alfred Psychiatry Research Centre, The Alfred Hospital and Monash University, Central Clinical School, Brain and Psychological Sciences Research Centre, Swinburne University of Technology, The University of Melbourne and St. Vincent’s Hospital

## Abstract

**Background:**

It has been reported that individuals with schizophrenia who are homozygous at the c.267C > A single nucleotide polymorphism (rs2067477) within the cholinergic muscarinic M1 receptor gene exhibit impaired Wisconsin Card Sorting Test (WCST) performance compared to those who are heterozygous. This investigation sought to examine the influence rs2067477 genotype variation has on general cognitive function.

**Methods:**

87 individuals with schizophrenia/schizoaffective disorder (Sz/SAD) and 224 healthy controls (HC) completed the MATRICS Consensus Cognitive Battery and D-KEFS Stroop to determine whether rs2067477 genotype variation influenced cognition.

**Results:**

No significant differences in MCCB domain scores or D-KEFS Stroop were found across genotype in both a patient-only sample and a combined patient-healthy control sample

**Discussion:**

Despite rs2067477 genotype variation being shown to influence executive functioning, specifically performance on the WCST in individual with schizophrenia, no such association could be detected across a number of general cognitive domains or on an alternative measure of shifting/cognitive flexibility.

